# Vitamin D deficiency in patients with cluster headache: a preliminary study

**DOI:** 10.1186/s10194-018-0886-7

**Published:** 2018-07-17

**Authors:** Jong-Hee Sohn, Min-Kyung Chu, Kwang-Yeol Park, Hong-Yup Ahn, Soo-Jin Cho

**Affiliations:** 1 Department of Neurology, Chuncheon Sacred Heart Hospital, Hallym University College of Medicine, Chuncheon-si, Gangwon-do Korea; 2 Department of Neurology, Severance Hospital, Yonsei University College of Medicine, Seoul, Korea; 3 Department of Neurology, Chung-Ang University Hospital, Chung-Ang University College of Medicine, Seoul, Korea; 4 Department of Statistics, Dongguk University-Seoul, Seoul, Korea; 5 Department of Neurology, Dongtan Sacred Heart Hospital, Hallym University College of Medicine, Keun Jae Bong-gil 7, Hwaseong, Gyeonggi-do 18450 Korea

**Keywords:** Vitamin D, Deficiency, Cluster headache, Migraine

## Abstract

**Background:**

Cluster headache is famous for attacks with seasonal and diurnal periodicity. This diurnal and seasonal variation might be related to sunlight and vitamin D metabolism. We investigated the serum vitamin D levels in patients with cluster headache.

**Methods:**

We enrolled patients with cluster headache and age- and sex-matched migraineurs and normal controls. From October 2016 to March 2018, non-fasting serum 25(OH)D concentrations were measured using a chemiluminescent immunoassay. Vitamin D deficiency was defined as a concentration < 20 ng/mL.

**Results:**

The study enrolled 28 patients with cluster headache, 36 migraineurs, and 36 normal controls. In the patients with cluster headache, the serum 25(OH)D concentration averaged 14.0 ± 3.9 ng/mL and 92.8% had vitamin D deficiency. There was no significant difference among the patients with cluster headache, migraineurs, and controls. In the patients with cluster headache, there was no difference in the serum 25(OH)D concentrations between men and women, cluster and remission periods, first and recurrent attack, presence and absence of daily or seasonal periodicity, and 3-month recurrence. In the 14 patients with seasonal periodicity, patients with periodicity of winter to spring had a trend of lower serum 25(OH)D concentrations than those with periodicity of summer to autumn (12.30 ± 1.58 vs. 16.96 ± 4.69 ng/mL, *p* = 0.097).

**Conclusions:**

Vitamin D deficiency is common in patients with cluster headache, but the role of vitamin D deficiency is uncertain, except for its seasonal influence.

**Electronic supplementary material:**

The online version of this article (10.1186/s10194-018-0886-7) contains supplementary material, which is available to authorized users.

## Background

Vitamin D is produced in the skin after oral absorption and exposure to sunlight. It is transformed by the liver and kidneys and plays a crucial role in promoting bone and muscle health. Vitamin D deficiency is a global health issue and is associated with inflammation, autonomic function, autoimmune disorders, chronic pain, cancer, and vascular and neurological disorders, in addition to its skeletal roles [1–6].

The estimated prevalence of vitamin D deficiency in North America, Europe and Australia is as high as 20–50% [7–13]. Vitamin D deficiency is more common in Korea (37° N) where the prevalence in male and female adult workers was 69.5 and 83.1%, respectively [14]. The determinants of vitamin D levels are the amount of skin exposure, followed by location, season, personal ultraviolet radiation exposure, vitamin D supplementation, body mass index, and physical activity [15].

A high prevalence of all kinds of headache has been associated with high latitude, suggesting a relationship with vitamin D [16]. However, the inter-relationships of vitamin D deficiency and headache are still uncertain. The studies of vitamin D in primary headache disorders have focused mainly on migraine, although there is no systematic evidence of an association between vitamin D deficiency and migraine [17–19]. There are case reports of migraine patients who improved with vitamin D supplementation [20, 21]. Simvastatin plus vitamin D has been reported to be effective for preventing headache in adults with episodic migraine in randomized clinical trials [22]. Dysfunction of vitamin D-binding protein and polymorphism of the vitamin D receptor gene have been reported in patients with migraine, while the relationship between the serum vitamin D level and headache frequency in migraine patients is controversial [23–25].

Tension-type headache is the most common primary headache disorder, accounting for most non-migraine headache in population studies. In the Norwegian population, non-migraine type headache was associated with low vitamin D levels with an odds ratio of 1.20 in the lowest quartile compared with the highest quartile group, whereas no significant association was found between migraine and the vitamin D levels [17]. There are also reports that chronic tension-type headache was associated with vitamin D deficiency [26–28].

Cluster headache shows a seasonal predilection, with nocturnal attacks [29–31]. The diurnal and seasonal variation might be related to sunlight and vitamin D metabolism, suggesting a role for vitamin D in cluster headache. There are no published studies of vitamin D in cluster headache, although a poster presentation showed that an anti-inflammatory regimen of vitamin and mineral supplements including vitamin D3 was effective in preventing cluster headaches [32]. Therefore, we investigated the serum vitamin D level in patients with cluster headache and the relationship between vitamin D deficiency and headache parameters compared with migraineurs and healthy controls.

## Methods

### Subjects

We enrolled patients with cluster headache and age- and sex-matched migraine patients as headache controls and normal controls in a case–control design. This study collected data from patients with cluster headache and migraine treated in the headache clinics of Chuncheon, Dongtan, and Kangnam Sacred Heart Hospitals of Hallym University College of Medicine from October 2016 to March 2018. All participants were between 20 and 65 years of age, and they were examined and classified according to the beta version of the International Classification of Headache Disorders-3 (ICHD-3β) [33] by a board-certified neurologist based on the patient history, a neurological examination, and neuroimaging studies. The control group consisted of volunteers matched for age, gender, and season enrolled. We recruited the control group via advertisements such as posted notices in the hospital. Control participants were required to be headache free (less than 1 headache day per month for an average of 1 year) with no history of primary or secondary headache disorder by the ICHD-3β [31, 33]. Exclusion criteria included subjects who took a vitamin D supplement or medication that influences vitamin D metabolism (e.g., antiepileptic drugs, rifampin, antiretroviral drugs, etc.), severe obesity [body mass index (BMI) > 30], thyroid or parathyroid disease, diabetes, and severe liver or renal disease.

The study protocol and informed consent form were reviewed and approved by the Institutional Review Board of each hospital (2016-439-I). Written informed consent was obtained from all participants before enrolling them in the study.

### Demographic data and questionnaire

Demographic data were obtained from all groups, including age, sex, height, weight, place of residence, occupation, level of education, and smoking habit. The data collected on headache patients included frequency of attacks (per month), average headache duration (hours), severity of pain [assessed with a visual analogue scale (VAS) range 0–10], and days per month using abortive medication. The patients with cluster headache completed a detailed semi-structured questionnaire on the headache characteristics, such as autonomic symptoms, diurnal periodicity, time of propensity, and seasonal periodicity. We contacted the patients after at least 3 months and collected data on the recurrence of headache. Eligible patients were interviewed at follow-up outpatient appointments or by telephone. The headache group completed the Headache Impact Test-6 (HIT-6) to measure headache-related impact, Patient Health Questionnare-9 (PHQ-9) to assess depression, Generalized Anxiety Dirorder-7 (GAD-7) to assess anxiety, and Pittsburgh Sleep Quality Index (PSQI) to measure the quality of sleep [34–37].

### Measurement and analysis of vitamin D

Non-fasting venous blood samples were collected at the baseline examinations. Serum 25(OH)D concentrations were measured with a chemiluminescent immunoassay using the ARCHITECT i4000 SR (Abbott Diagnostics, IL, USA). The intra- and interassay coefficients of variation of the measurements were 1.7–2.8 and 2.7–4.1%, respectively. The assay was standardized against NIST Standard Reference Material 2972 (NIST, Gaithersburg, ME, USA) and certified by the Centers for Disease Control and Prevention Vitamin D Standardization Program [38]. Vitamin D deficiency was defined as a 25(OH)D < 20 ng/mL, insufficiency as 21–29 ng/mL, and ideal range as 30–50 ng/mL.

The time of the day, month, and year when the vitamin D assessment was performed was recorded [39, 40]. Most experts define vitamin D deficiency as serum 25(OH)D vitamin D levels < 20 ng/mL, although there is no consensus on the optimal serum vitamin D levels [41–44]. A cutoff value of 20 ng/mL was used to divide the samples into “deficient” and “normal” groups in this study [14].

### Statistics

Analysis of variance (ANOVA) was used to compare demographic and headache parameters among groups, after confirming the normality of the distribution using the Shapiro-Wilk test. If the normality of the distribution was not confirmed, the Kruskal-Wallis, chi-square test or Fisher’s exact test were used to compare data between groups, where appropriate. Serum vitamin D concentrations in subjects with CH, migraine, and controls were compared using analysis of covariance (ANCOVA), with adjustment for potential confounders. The correlations between the serum 25(OH)D concentration and various clinical features were assessed using Pearson’s or Spearman correlation coefficient based on the normality of the distribution.

To compare vitamin D concentration according to the headache parameters among the CH group, the Mann-Whitney test was used. ANOVA was used to compare vitamin D concentrations between subgroups in CH patients. Using linear regression analysis, we calculated the odds ratios of vitamin D levels associated with the headache variables. The variables that showed statistical correlations with the vitamin D levels in the univariate analyses, and factors that are known to affect the vitamin D level, were included in the multivariate regression analysis. Linear regression was used to assess the seasonal propensity of serum 25(OH)D concentrations with factors known to affect vitamin D levels (age, male, current smoker, sampling time) [45]. A *p*-value < 0.05 was considered statistically significant. All analyses were performed using R version 3.5.0 for Windows (R Foundation for Statistical Computing, Vienna, Austria. http://www.r-project.org/) and RStudio version 1.1.442.

## Results

### Comparison of demographics and vitamin D levels in patients with cluster headache, migraine, and controls

The study enrolled 28 patients with cluster headache, 36 migraineurs, and 36 normal controls. No significant differences were found in the CH and migraine patients compared with the controls in terms of age (38.2, 35.1, and 35.4 years, respectively) or sex (proportion male 85.7, 80.6, and 88.9%, respectively). In addition, there were no differences in BMI, smoking status, sampling season, and sampling time zones (7-10 AM, 11 AM-1 PM, 2-5 PM) among the three groups (Table 1).

**Table 1:** Demographic features and vitamin D parameters of the patients with cluster headache, migraineurs, and normal controls

	Cluster Headache (*n* = 28)	Migraine (*n* = 36)	Normal controls (*n* = 36)	*p*-value
Age, years	38.2 ± 8.5	35.1 ± 8.2	35.4 ± 9.1	0.189
Men (%)	24 (85.7)	29 (80.6)	32 (88.9)	0.607
BMI, kg/m^2^	23.3 ± 2.8	23,6 ± 3.7	24.1 ± 3.2	0.583
Smoking habit (%)^a^				0.133
Current	17 (63.0)	15 (41.7)	14 (38.9)	
ex-smoker	3 (11.1)	2 (5.6)	8 (22.2)	
Never	7 (25.9)	19 (52.8)	14 (38.9)	
Pain intensity, VAS	8.5 ± 1.8	6.4 ± 1.7	NA	< 0.001
Vitamin D deficiency (%)	26 (92.8)	29 (80.6)	30 (83.3)	0.771
Distribution of 25(OH)D concentration				0.236
≥ 30 (ng/mL)	0	1 (2.8)	2 (5.6)	
20–29.9 (ng/mL)	2 (7.2)	6 (16.7)	4 (11.1)	
10–19.9 (ng/mL)	23 (82.1)	21 (58.3)	19 (52.8)	
< 10 (ng/mL)	3 (10.7)	8 (22.2)	11 (30.5)	
Sampling seasons				0.349
Winter to spring	15 (53.9)	21 (58.3)	15 (41.7)	
Summer to autumn	13 (46.4)	15 (41.7)	21 (58.3)	

Values are expressed as mean ± standard deviation or number (%)

*VAS* visual analogue scale

^a^data was not available in one patient with CH

The serum 25(OH)D concentrations were 14.0 ± 3.9 ng/mL in the patients with CH, 14.7 ± 5.9 ng/mL in the migraineurs, and 14.6 ± 7.4 ng/mL in the normal controls (*p* = 0.884). After adjustment for potential confounders (sex, age, sampling season, current smoking) by ANCOVA, the serum 25(OH)D concentrations were not different between groups (data not shown, *p* = 0.853). Vitamin D deficiency was present in 92.8% of the patients with CH, 80.6% of the migraineurs, and 83.3% of the normal controls (*p* = 0.771) There were no significant differences in distribution of serum 25(OH)D concentrations between patients with CH, migraine, and controls (Table 1).

Serum 25(OH)D concentrations were higher in those with a sampling season from summer to autumn than those with a sampling season from winter to spring (15.6 ± 5.2 ng/mL vs. 13.3 ± 6.5 ng/mL, *p* = 0.003). No differences in serum vitamin D concentrations were evident based on the time of blood sample collection when analyzed at one-hour intervals (data not shown). Furthermore, there were no correlations between serum 25(OH)D concentrations and age, BMI, GAD-7 score, or PHQ-9 score, nor were any differences of the level observed between sexes.

### Comparison of vitamin D levels in the patients with cluster headache according to clinical factors

In patients with CH, there was no difference in the serum 25(OH)D concentrations between men and women, cluster and remission periods, first and recurrent attack, presence and absence of daily or seasonal periodicity, and 3-month recurrence (*p* > 0.05). In the 14 patients with seasonal periodicity, patients with periodicity of winter to spring had a trend toward lower serum 25(OH)D concentrations than those with periodicity of summer to autumn (12.30 ± 1.58 vs. 16.96 ± 4.69 ng/mL, *p* = 0.097, Table 2).

**Table 2:** Vitamin D levels according to clinical variables in the patients with cluster headache

	25(OH)D, ng/mL	*p*-value
Sex		0.646
Men (*n* = 24)	13.95 ± 4.08	
Women (*n* = 4)	14.33 ± 2.82	
Recurrence		0.300
First episode (*n* = 6)	14.83 ± 2.42	
Recurrent episode (*n* = 22)	13.78 ± 4.21	
Paired comparison		0.421
Cluster period (*n* = 14)	13.87 ± 3.64	
Remission period (*n* = 14)	14.06 ± 4.56	
Diurnal periodicity		0.728
Present (*n* = 16)	13.69 ± 3.47	
Absent (*n* = 12)	14.41 ± 4.50	
Time of propensity		0.125
Nighttime propensity (*n* = 9)	14.72 ± 3.63	
Daytime propensity (*n* = 7)	11.71 ± 2.63	
Recurrence at 3 months		0.562
present (*n* = 2)	13.00 ± 3.39	
absent (*n* = 26)	14.08 ± 3.97	
Seasonal periodicity		0.188
Present (*n* = 14)	14.63 ± 4.14	
Absent (*n* = 8)	12.29 ± 4.18	
Season of cluster period		0.097
Winter to spring (*n* = 7)	12.30 ± 1.58	
Summer to autumn (*n* = 7)	16.96 ± 4.69	
Sampling season		0.001
Winter to spring (*n* = 15)	11.91 ± 2.63	
Summer to autumn (*n* = 13)	16.41 ± 3.76	

Vitamin D levels were significantly lower in the subgroup with sampling season between winter to spring relative to other groups. Therefore, we analyzed vitamin D levels in the CH subgroups according to seasonal propensity, and by sample season for the 22 CH patients with recurrence (Table 3). There were significant differences in vitamin D levels between six groups; however, the differences between sampling seasons were not significant. There was no correlation between the vitamin D level and daily frequency, average weeks of cluster period, total bout of cluster period, or HIT-6, GAD-7, or PHQ-9 score.

**Table 3:** The 25(OH)D concentration according seasonal propensity and sample seasons in 22 CH patients with recurrence

According to sample seasons	Seasonal propensity		No seasonal propensity (*n* = 8)	*p*-value
	Winter to spring (*n* = 7)	Summer to autumn (*n* = 7)		
Winter to spring (*n* = 12)	12.28 ± 1.93^a^ (*n* = 5)	13.00 ± 3.39^c^ (*n* = 2)	9.48 ± 1.61^e^ (*n* = 5)	0.001
Summer to autumn (*n* = 10)	12.35 ± 0.07^b^ (*n* = 2)	18.54 ± 4.38^d^ (*n* = 5)	16.97 ± 1.82^f^ (*n* = 3)	

One-way analysis of variance (ANOVA) was done based on normal distribution of vitamin D level among 28 patients with cluster headache. According to post-hoc analysis between 6 groups using Turkey HSD, *p*-values between a and d was 0.023, *p*-value between d and e was < 0.001, and *p*-value between e and f was 0.018. The other comparisons were not significant

*CH* cluster headache

### Clinical factors associated with vitamin D levels

The univariate linear analysis showed that sampling from summer to autumn and seasonal propensity for summer to autumn showed significant correlations with the vitamin D level. In the multiple linear regression analysis, however, the significance of seasonal propensity for summer to autumn was lost after adjusting for the sampling season. No changes in significance were observed by the multiple linear regression analysis when controlling for age, current smoking status, and sampling time zone (Additional file 1: Table S1).

## Discussion

This study suggested that vitamin D deficiency is very common in patients with cluster headache compared with the prevalence of vitamin D deficiency in Korean adults [14]. However, the significance of vitamin D deficiency is uncertain, except for a subtle seasonal influence.

A few recent observational studies suggested that low serum vitamin D levels were related to some headache disorders, especially migraine and tension-type headache [24, 28, 46, 47]. One study also reported significant relations of lifetime prevalence of both migraine and tension-type headache with latitude [16]. If vitamin D has any association with headache, the prevalence of headache should match the seasonal variation in vitamin D levels. There are data indicating an increased frequency of headache attacks in autumn–winter and the least number of attacks in summer, although there are few studies on the seasonal variation of primary headache disorders, such as migraine and tension-type headache [48–50].

However, the relationship between cluster headache and vitamin D has not been studied previously, although cluster headache has characteristic seasonal rhythmicity. Cluster headache is a primary headache disorder characterized by a unique seasonal and diurnal periodicity. These characteristic features of its periodicity suggest involvement of the hypothalamus, the biological clock [51]. In Korea, seasonal propensity and diurnal periodicity were present in 44.0 and 68.5% of the patients with cluster headache, respectively. Among patients with seasonal propensity and diurnal periodicity, the most frequently cited season and time was spring (37.5%) and night (66.4%), respectively [52]. An inverse relationship between sunshine duration and the monthly incidence of cluster periods was reported in a hospital-based study [53]. A follow-up population-based study found positive correlations between average temperature and sunshine duration with occurrence of cluster periods, and that temperature was associated with precipitating or priming cluster periods [30]. Based on previous results, it is possible that vitamin D and cluster headaches are related. However, our study did not find significant differences in the vitamin D level among cluster headache, migraine, and controls. Although there was no significant difference among groups, the prevalence of vitamin D deficiency was higher in the cluster headache group than in the migraine and control groups. This suggests that vitamin D deficiency is very common in patients with cluster headache compared with the prevalence of vitamin D deficiency in Korean adults [14]. There was also no significant difference in vitamin D levels according to clinical variables in the patients with cluster headache. However, patients with periodicity of winter to spring had a trend of lower serum 25(OH)D concentrations than those with periodicity of summer to autumn in patients with seasonal periodicity. The serum vitamin D level varies with the season, peaking in May to September. The levels tend to be lower from November to March [54–56]. Therefore, we used an arbitrary classification of the sunny season, i.e., summer to autumn, winter to spring.

The vitamin D receptor and 1α-hydroxylase, the enzyme responsible for the formation of the active vitamin, are present in many parts of the central nervous system, including the prefrontal cortex, hippocampus, cingulate gyrus, thalamus, and hypothalamus. The strongest immunohistochemical staining for both the receptor and enzyme was in the hypothalamus [57]. Vitamin D-binding protein was also detected in axonal projections throughout the lateral hypothalamus in the rat [58]. The presence of the vitamin D receptor, 1α-hydroxylase, and vitamin D-binding protein in the hypothalamus suggests a role of vitamin D deficiency in the generation of various primary headache disorders. In addition to the hypothalamus, there is evidence that the pineal gland has a role in the biological regulation of circadian rhythms by producing melatonin [59]. Sunshine has been reported to regulate specific hormonal and neurotransmitter levels, including melatonin [60]. Altered melatonin levels have been documented in cluster headache and the nocturnal serum melatonin level was decreased during cluster periods [61]. Based on the characteristic clinical features and proposed mechanisms of cluster headache, we hypothesized that vitamin D and cluster headache are related. However, our study failed to find any correlations between the serum vitamin D levels and headache characteristics, such as the seasonal and circadian rhythmicity of cluster headache or the association between serum vitamin D levels and recurrence of cluster headache. To determine the associations between vitamin D and the recurrence in cluster headache, a further longitudinal follow-up study of a larger population is required.

Various factors influence serum vitamin D levels, making, it is difficult to speculate on the role of vitamin D in the pathophysiology of cluster headache. Our study could not control for all of the environmental and demographic factors that significantly affect the serum vitamin D levels There was no information on the exposure of the subjects to sunlight, their sun protection habits or the extent of vitamin D supplementation through food, though we did collect information on smoking status, BMI, sampling time, and sampling season, all of which are known to influence vitamin D concentration. We also analyzed the influence of these variables on serum vitamin D levels. There were no significant differences in smoking status and BMI among the three groups. Additionally, current smoking was not a significant determinant of serum vitamin D levels in this study population using multivariate regression analysis. Second, our study had a relatively small sample size due to the rarity of cluster headache. This study suggests that the influence of vitamin D may warrant analysis in a larger cohort of CH patients with seasonal propensity for winter to spring. This study also included mostly men, due to a sex-matched research design, so these findings may not be truly representative of migraine and normal controls, or generalizable to other groups. Third, the outcome variables collected, such as the seasonal propensity, were assessed on self-report by recall and the classification of the sunny season was arbitrary. While we did not obtain fasting serum samples, there were no significant differences in sampling times among the three groups based on the time the blood samples were collected.

## Conclusions

In summary, this is the first report to examine serum vitamin D levels in patients with CH compared to those of age-and sex-matched controls controlling for sampling season as well as other clinical variables.. Our preliminary findings suggest that vitamin D deficiency is very common in patients with cluster headache, but the significance of vitamin D deficiency is uncertain, except for the subtle seasonal influence. Further research is needed to determine the role of vitamin D in CH.

## Additional file

Additional file 1: **Table S1.** Coefficients of linear regression analysis of the vitamin D levels in 22 CH patients with recurrence. (DOCX 14 kb)

## Abbreviations

BMI: Body mass index
GAD-7: Generalized Anxiety Dirorder-7
HIT-6: Headache Impact Test-6
ICHD-3β: Beta version of the International Classification of Headache Disorders-3
PHQ-9: Patient Health Questionnare-9
PSQI: Pittsburgh Sleep Quality Index
SE: Standard error
VAS: Visual analogue scale
