# Supplementary material for: Vitamin D deficiency in patients with cluster headache: a preliminary study
Source: J Headache Pain. 2018 Jul 17;19(1):54. doi: 10.1186/s10194-018-0886-7 (PMC6049846; doi:10.1186/s10194-018-0886-7)
Supplement: Supplementary file 1 — Table S1. Coefficients of linear regression analysis of the vitamin D levels in 22 CH patients with recurrence. (DOCX 14 kb) [file 10194_2018_886_MOESM1_ESM.docx]

Additional file 1: **Table S1.** Coefficients of linear regression analysis of the vitamin D levels in 22 CH patients with recurrence.

|  | Univariate | | Model 1 | | Model 2 | |
| --- | --- | --- | --- | --- | --- | --- |
|  | *B* (SE) | *p-*value | *B* (SE) | *p-*value | *B* (SE) | *p-*value |
| Sampling season |  |  |  |  |  |  |
| winter to spring | 1 |  | 1 |  | 1 |  |
| summer to autumn | 5.60 (1.36) | <0.001 | 4.66 (1.40) | 0.037 | 4.91 (1.85) | 0.022 |
| Seasonal propensity |  |  |  |  |  |  |
| absent | 1 |  | 1 |  | 1 |  |
| winter to spring | 0.01 (1.95) | 0.995 | 0.42 (1.58) | 0.789 | 0.75 (2.13) | 0.730 |
| summer to autumn | 4.67 (1.95) | 0.027 | 3.09 (1.64) | 0.077 | 2.93 (2.30) | 0.227 |

*B,* unstandardized beta; SE, standard error; Model 1 was analyzed with sampling season and seasonal propensity.
